# Prognostic Significance of Tag SNP rs1045411 in *HMGB1* of the Aggressive Gastric Cancer in a Chinese Population

**DOI:** 10.1371/journal.pone.0154378

**Published:** 2016-04-26

**Authors:** Guoqiang Bao, Falin Qu, Li He, Huadong Zhao, Nan Wang, Gang Ji, Xianli He

**Affiliations:** 1 Department of General Surgery, Tangdu Hospital, The Fourth Military Medical University, Xi’an, 710032, China; 2 Department of Ophthalmology, School of Medicine, Emory University, Atlanta, GA 30322, United States of America; 3 Xijing Hospital of Digestive Disease, The Fourth Military Medical University, Xi'an, 710032, China; Duke Cancer Institute, UNITED STATES

## Abstract

Compelling evidences have suggested that high mobility group box-1 (HMGB1) gene plays a crucial role in cancer development and progression. This study aimed to evaluate the effects of single nucleotide polymorphisms (SNPs) in *HMGB1* gene on the survival of gastric cancer (GC) patients. Three tag SNPs from *HMGB1* gene were selected and genotyped using Sequenom iPEX genotyping system in a cohort of 1030 GC patients (704 in training set, 326 in validation set). Multivariate Cox proportional hazard model and Kaplan-Meier Curve were used for prognosis analysis. AG/AA genotypes of SNP rs1045411 in *HMGB1* gene were significantly associated with better overall survival (OS) in a set of 704 GC patients when compared with GG genotypes (HR = 0.77, 95% CI: 0.60–0.97, *P* = 0.032). This prognostic effect was verified in an independent validation set and pooled analysis (HR = 0.80, 95% CI: 0.62–0.99, *P* = 0.046; HR = 0.78, 95% CI: 0.55–0.98, *P* = 0.043, respectively). In stratified analysis, the protective effect of rs1045411 AG/AA genotypes was more prominent in patients with adverse strata, compared with patients with favorable strata. Furthermore, strong joint predictive effects on OS of GC patients were noted between rs1045411 genotypes and Lauren classification, differentiation, stage or adjuvant chemotherapy. Additionally, functional assay indicated a significant effect of rs1045411 on *HMGB1* expression. Our results suggest that rs1045411 in *HMGB1* is significantly associated with clinical outcomes of Chinese GC patients after surgery, especially in those with aggressive status, which warrants further validation in other ethnic populations.

## Introduction

Gastric cancer (GC) is the fourth most common cancer in the world, accounting for about 8% of new cancers and 10% of cancer deaths [1]. Of these cases, 70% occurred in developing countries, and half of the world total occurred in Eastern Asia, predominantly in China [2]. Over the past few decades, despite the significant increase in the investment and advances in the diagnosis and treatment of GC, the overall survival (OS) for advanced GC is still dismal, with a 5-year survival rate of less that 25% [3]. Currently, the survival and prognosis of GC patients still depend on the stage of the tumor at the time of diagnosis. However, due to the clearly important differences within the same stage, tumor stage alone is not sufficient to predict the prognosis of GC [4]. Therefore, to discover novel molecular signatures as reliable prognostic markers for GC is very important and demanding. In recent years, studies have focused on the investigation of genetic variants that predispose to the development and progression of GC [5].

High mobility group box-1 (HMGB1), an important member of high-mobility group protein superfamily, contains two 80-amino acid DNA-binding domains (A-box and B-box) and an acidic carboxyl tail [6]. It functions as a chromatin structural protein within the nucleus and a proinflammatory cytokine extracellularly. As a nuclear protein, HMGB1 binds non-specifically to the minor groove of DNA and facilitates the assembly of site-specific DNA targets [7]. In contrast, extracellular HMGB1 functions as a cytokine that propagates infection- or injury-elicited inflammatory responses [8]. The constant release of HMGB1 from necrotic tumor cells may create a microenvironment resembling chronic inflammation; a condition known to contribute to the development of epithelial malignancies, especially inflammation-associated cancer [9]. In fact, numerous studies have previously demonstrated the over-expression of HMGB1 in many types of cancer [10–13], including GC [14]. Moreover, compelling evidences have further confirmed that HMGB1 over-expression is closely related to tumor development by mediating the proliferation, invasion and migration of cancer cells [15, 16]. Therefore, HMGB1 may be an interesting candidate as a novel prognostic marker or therapeutic target for GC.

Accumulating evidences have suggested that genetic backgrounds may affect the risk and prognosis of GC [17]. Single nucleotide polymorphism (SNP) is the most common genetic variation, and may be the promising surrogate biomarkers of patients’ genetic backgrounds to predict therapeutic response and prognosis [18]. Genetic variants have been identified in human *HMGB1* gene [19], but the association between *HMGB1* gene polymorphism and GC survival outcome has never been determined. Given the crucial role of HMGB1 in the development and progression of cancer, it is plausible that the polymorphisms of *HMGB1* may affect the clinical outcomes of GC. Herein, we assessed the effects of three tag SNPs in *HMGB1* on clinical outcomes of 1030 Chinese GC patients (704 in the training set, 326 in the independent validation set) who received radical resection treatment. Additionally, the effect of an identified relevant tag SNP on the regulation of gene expression was further examined by an *in vitro* functional assay. To the best of our knowledge, this is the first investigation of the association between the polymorphisms of *HMGB1* and the clinical outcome of GC.

## Materials and Methods

### Ethics

This study was approved by the Ethic Committee of The Fourth Military Medical University. The procedures were performed according to the approved guidelines and to the 1964 Helsinki Declaration and its later amendments or comparable ethical standards. The signed informed consent was obtained from each participant included in the study.

### Study population

A total of 1030 Han Chinese patients with primary gastric adenocarcinoma were enrolled from two independent sites, Tangdu Hospital and Xijing Hospital of Digestive Disease, in Xi’an, China. All GC cases received surgical resection and had no previous history of other cancers or any preoperative anticancer treatment or blood transfusion within 3 month before surgery. There were no age, sex, or disease stage restrictions for case recruitment. Among them, the 704 patients (Department of General Surgery, Tangdu Hospital, between July 2008 and June 2013) were used as a training set in this study. Another group of 326 patients (Xijing Hospital of Digestive Disease, between January 2008 and December 2010) were used as an independent validation set. The aim was to identify the clinically significant prognostic value of SNPs within *HMGB1* gene from the training set and tested it in the independent validation set.

### Demographic and clinical data

Demographic and clinical data were collected through in-person interviews at the initial visit or follow-up in the clinics, medical records, or consultation with treating physicians, including age, sex, ethnicity, residential region, time of diagnosis, time of surgery and/or adjuvant chemotherapy (ACT), time of relapse and/or death, tumor stage, Lauren classification, differentiation, histological type, and treatment protocol. Follow-up information was updated at 6-month intervals through on-site interview, telephone communication, or reviewing medical records by trained research specialists. The latest follow-up date was June 2015 and the median follow-up duration was 51 months (range 6–89 months). The percentage of patient lost during follow-up was 9.8%. OS was defined as the time from surgery to GC-specific death. RFS (Recurrence-free survival) was defined as the time from surgery to the date of the first recurrence or distant metastasis of GC. Patients alive at the last follow-up were censored.

### Collection, processing and preservation of specimens

Before surgery, 5 ml venous blood was collected from each GC patients to extract DNA using the E.Z.N.A. blood DNA Midi Kit (Omega Bio-Tek, Norcross, GA, USA). Sixty gastric cancerous tissues were simultaneously gathered from the validation set for real-time quantitative reverse transcription PCR (RT-PCR) assays.

### SNP selection and genotyping

The candidate tag SNPs selection of *HMGB1* gene were performed as a two-step procedure. Firstly, we used a set of web-based SNP selection tools (http://snpinfo.niehs.nih.gov/snpfunc.htm) to search candidate SNPs of *HMGB1* [20]. All validated polymorphisms in *HMGB1* gene region, including 5 kb upstream of the first exon and 5 kb downstream of the last exon, were considered to be candidate SNPs. Those SNPs with a minor allele frequency ≥ 5% in the HapMap CHB (Han Chinese in Beijing) population and a pairwise linkage disequilibrium squared correlation coefficient (r^2^) > 0.8 were selected as candidate SNPs. Secondly, tag SNPs were chosen from these candidate SNPs using the International HapMap Project Phase II database of the Chinese population (http://www.hapmap.org/, accessed 18 November 2013) and HAPLOVIEW version 4.2. Finally, three tag SNPs (mean r^2^ = 0.981) were selected: rs1045411(G>A), rs1412125(T>C) and rs2249825(C>G).Genotyping was carried out using Sequenom iPLEX genotyping system (Sequenom Inc., San Diego, CA, USA) according to the manufacturer’s protocol. The laboratory persons who conducted the genotyping assays were blinded to patients’ information. Internal quality controls and negative controls were used to ensure genotyping accuracy, and 5 samples were randomly selected and genotyped in duplicate with 100% concordance. Call rate for genotyping ranged between 99.3% and 99.7%. The detailed information of SNPs and genotyping results were listed in S1 Table.

### Functional assay

The functional effects of tag SNP rs1045411 located in the 3’UTR of *HMGB1* gene were investigated through using the luciferase reporter assay. Briefly, 49-bp double-strand DNA carrying either wild genotype or variant genotype of rs1045411 was synthesized and cloned into the pMIR-REPORT vector (Ambion, Austin, Tex, USA) using restriction enzymes Spe I and Hind III (Takara, Dalian, China). All the constructs were confirmed by DNA sequencing. Human GC cell lines SGC-7901 and human embryonic kidney cell line HEK-293T, in which has-miR-505 was indentified to be positively expressed by using TaqMan microRNA Reverse Transcription kit (Applied Biosystem, Foster City, CA, USA) as previously described [21], were co-transfected with either pMIR-rs1045411-A or pMIR-rs1045411-G (200ng/well) with or without anti-miR-505 (Applied Biosystems, Foster City, CA, USA) and the internal control reporter plasmid pRLTK (Promega, Madison, WI, USA) (20 ng/well) using Lipofectamine 2000 (Invitrogen, Carlsbad, CA, USA) in a 24-well plate with 2×10^5^ cells per well. SGC-7901 and HEK293 cell lines were purchased from the Type Culture Collection of the Chinese Academy of Sciences (CAS) (Shanghai, China), where they were verified by mycoplasma detection, DNA-Fingerprinting, isozyme detection and cell vitality detection. A frozen vial of each 147 cell line, which was immediately expanded and frozen when being received from the vendor, was resuscitated and used for the present study. After 48 h, the cells were collected to determine luciferase activity using a dual-luciferase reporter assay system kit (Promega, Madison, WI, USA) with a luminometer(Tecan, Mannedorf, Switzerland). All transfections were performed in triplicates, and all experiments were independently repeated three times.

To further assess the effect of tag SNP rs1045411 genotypes on the expression of *HMGB1* mRNA, total RNA was isolated from 60 GC tissue samples (30 with AA genotype and 30 with AG/GG genotypes of rs1045411) in accordance with the manufacturer’s instructions. Then, cDNA were synthesized using PrimeScript RT reagent kit (Takara, Dalian, China). RT-PCR was performed using the following *HMGB1* primers: forward, 5’-TAAGAAGCCGAGAGGCAAAA-3’; reverse, 5’-AGGCCAGGATGTTCTCCTTT- 3’, and β-actin was used as an internal control (primers: forward, 5’-AAGACGTACTCAGGCCATGTCC-3’; reverse, 5’-GACCCAAATGTCGCAGTCAG-3’) [13]. Relative expression of *HMGB1* mRNA levels was determined using the relative quantification method and 2^-ΔΔCt^ analysis.

### Statistical analysis

Statistics analyses were carried out using the IBM SPSS Statistics 19.0 software (IBM). Normally distributed continuous variables were expressed as mean ± SD, while abnormally distributed continuous variables were expressed as median and range. Pearson’s χ^2^-test was used to test the differences of categorical variables. The difference of normally distributed continuous variables between two groups was analyzed by using Student’s *t*-test, while Mann-Whitney U test was employed for the comparison of abnormally distributed continuous variables. Multivariate Cox proportional hazard regression model was applied to assess the effect of individual SNP and patients’ characteristics on OS or RFS. Hazard ratios (HRs) and 95% confidence intervals (CIs) were estimated with adjustment for age, sex, Lauren classification, differentiation, TNM stage and ACT. Kaplan-Meier curves and log-rank tests were also used to evaluate effect of the individual SNPs on survival time. Statistics significance was set at a level of 0.05 and all *P* values reported in this study were two sided.

## Results

### Distribution of patients’ characteristics and prognosis analysis

GC patients’ characteristics was summarized in the S2 Table. Due to the late ending date of patient enrollment for the training set, the median follow-up time was shorter in the training set (46 months, ranging from 6 to 80 months) than that in the independent validation set (72 months, ranging from 6 to 89 months). Thus compared with the patients in the independent validation set, those in the training set had lower rates of relapse (58.4% *vs*. 66.8%, *P* = 0.005) and death (41.4% *vs*. 55.8%, *P* = 0.001). There were no differences between training set and validation set in terms of age, tumor site, Lauren classification, TNM stage, differentiation and ACT (*P* value ranging from 0.082 to 0.898). At latest follow-up, 641 patients (423 and 218 in the training and validation set, respectively) developed relapse and 482 died (300 and 182 in the training and validation set, respectively). Multivariate Cox regression analysis showed that there were significant higher death and recurrence risk in patients with diffuse type, poor differentiation and tumor stage III and IV compared with those patients with intestinal type, well/moderate differentiation and tumor stage I and II among training set, validation set and pooled analysis. In addition, platinum-based ACT after surgery showed a significant protective effect on both OS and RFS of GC patients (S3 Table).

### Association of *HMGB1* SNPs with clinical outcome in GC patients

**Fig 1:**
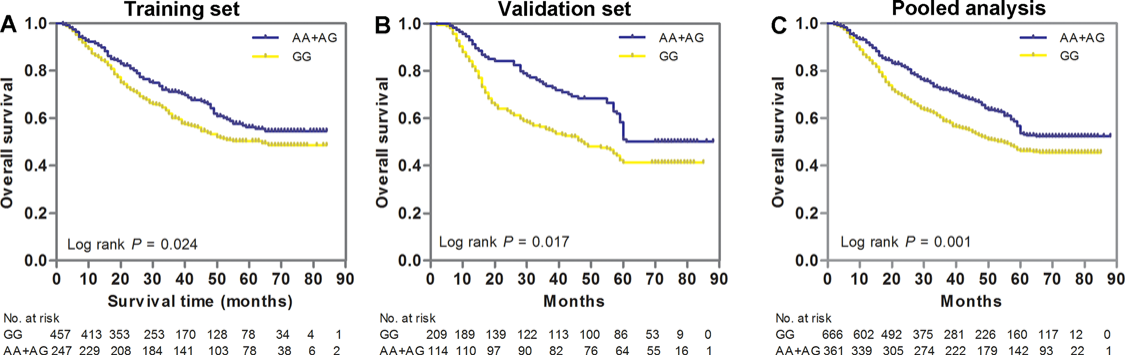
Kaplan-Meier estimates of overall survival (OS) for gastric cancer (GC) patients stratified by genetic variants of *HMGB1* gene. OS of GC patients stratified by SNP rs1045411 in the training set (A), validation set(B) and pooled analysis(C). Patient numbers may not add up to 100% of available subjects because of missing genotyping data.

We assessed the association of *HMGB1* SNP genotypes with GC clinical outcome using the multivariate Cox regression analysis with adjustment for age, sex, tumor site, Lauren classification, differentiation, TNM stage and ACT (as shown in Table 1). The results showed that tag SNP rs1045411 was significantly associated with OS of GC patients in the training set. Compared to patients with the GG genotype, those with variant alleles (AG and AA genotypes) had a significantly lower death risk (HR = 0.77, 95%CI: 0.60–0.97, *P* = 0.032). This significant finding was confirmed in the independent validation set and pooled analysis, with HRs of 0.80 (95% CI: 0.62–0.99; *P* = 0.046) and 0.78 (95% CI: 0.55–0.98; *P* = 0.043), respectively. Kaplan-Meier curves analysis also provided a strong association with OS. Patients carrying AG/GG genotypes of rs1045411 had better OS than did those with GG genotype in training set (*P* = 0.024, Fig 1A), validation set (*P* = 0.017, Fig 1B) and pooled analysis (*P* = 0.001, Fig 1C).

**Table 1:** Association of HMGB1 SNPs and clinical outcome of gastric cancer patients.

SNP	Genotype	Training set			Validation set			Pooled analysis		
		Events^a^/Total	HR^b^ (95% CI)	*P*^c^	Events^a^/Total	HR^b^ (95% CI)	*P*^c^	Events^a^/Total	HR^b^ (95% CI)	*P*^c^
Overall survival										
rs1045411	GG	201/457	Reference		123/209	Reference		324/666	Reference	
	AG	86/222	0.76 (0.59–0.99)	0.048	49/102	0.82 (0.60–1.06)	0.117	135/324	0.79 (0.54–1.04)	0.102
	AA	13/25	0.79 (0.41–1.55)	0.495	7/12	0.89 (0.57–1.68)	0.389	20/37	0.86 (0.43–1.51)	0.217
	AG/AA		**0.77 (0.60–0.97)**	**0.032**		**0.80 (0.62–0.99)**	**0.046**		**0.78 (0.55–0.98)**	**0.043**
rs2249825	CC	204/503	Reference		123/233	Reference		327/736	Reference	
	CG	85/182	1.23 (0.89–1.94)	0.364	52/83	1.29 (0.71–1.76)	0.412	137/265	1.22 (0.83–1.61)	0.223
	GG	6/14	1.06 (0.67–1.52)	0.681	5/8	1.18 (0.55–1.63)	0.771	11/22	1.11 (0.60–1.65)	0.753
	CG/GG		1.28 (0.90–1.88)	0.211		1.39 (0.73–2.15)	0.289		1.30 (0.88–1.72)	0.218
rs1412125	TT	155/375	Reference		94/173	Reference		249/548	Reference	
	CT	121/283	1.04 (0.81–1.34)	0.751	74/131	1.13 (0.54–1.62)	0.841	195/414	1.07 (0.63–1.48)	0.756
	CC	19/41	0.99 (0.58–1.69)	0.970	12/20	1.08 (0.66–1.49)	0.798	31/61	1.02 (0.60–1.52)	0.631
	CT/CC		1.04 (0.81–1.32)	0.780		1.17 (0.70–1.63)	0.800		1.09 (0.76–1.55)	0.679
Recurrence-free survival										
rs1045411	GG	280/457	Reference		142/209	Reference		422/666	Reference	
	AG	124/222	0.81 (0.63–1.04)	0.102	63/102	0.86 (0.54–1.128)	0.239	187/324	0.82 (0.58–1.09)	0.179
	AA	19/25	0.96 (0.52–1.76)	0.894	10/12	0.88 (0.48–1.49)	0.649	29/37	0.89 (0.46–1.55)	0.400
	AG/AA		0.82 (0.65–1.05)	0.118		0.84 (0.49–1.18)	0.149		0.83 (0.62–1.13)	0.198
rs2249825	CC	289/503	Reference		148/233	Reference		437/736	Reference	
	CG	121/182	1.29 (0.86–1.79)	0.292	62/83	1.32 (0.77–1.92)	0.413	183/265	1.37 (0.92–1.65)	0.183
	GG	9/14	1.08 (0.74–1.46)	0.795	6/8	1.25 (0.70–1.69)	0.762	15/22	1.19 (0.69–1.54)	0.684
	CG/GG		1.32 (0.91–1.95)	0.288		1.35 (0.83–1.99)	0.396		1.38 (0.94–1.79)	0.174
rs1412125	TT	218/375	Reference		121/173	Reference		339/548	Reference	
	CT	174/283	1.08 (0.85–1.38)	0.505	80/131	1.07 (0.61–1.57)	0.463	254/414	1.09 (0.55–2.18)	0.983
	CC	27/41	1.20 (0.75–1.94)	0.451	14/20	1.24 (0.81–1.91)	0.315	41/61	1.21 (0.63–2.45)	0.697
	CT/CC		1.10 (0.87–1.38)	0.420		1.09 (0.58–1.97)	0.714		1.14 (0.61–1.88)	0.513

Note: HR indicates hazard ratio; CI, confidence interval.

^a^ Numbers may not add up to 100% of available subjects because of missing genotyping data.

^b^ Adjusted by age, sex, tumor site, Lauren classification, differentiation, TNM stage, and chemotherapy where appropriate.

^c^ The significant values were shown in boldface (*P* < 0.05).

### Stratified analysis on association of rs1045411 with OS by host variables

**Fig 2:**
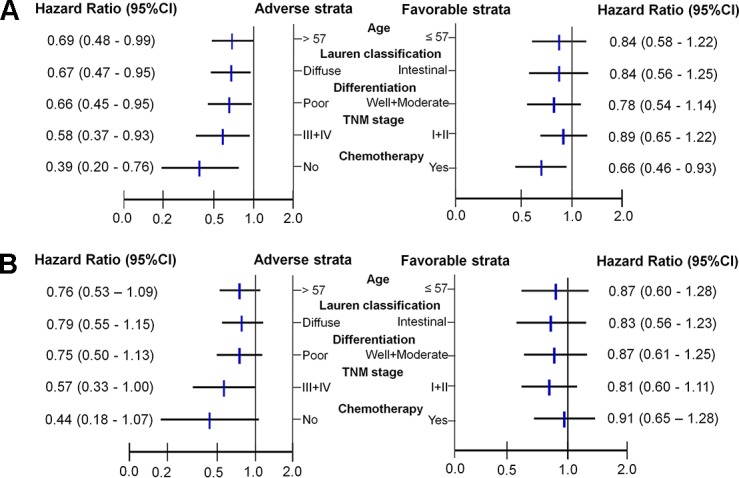
Stratified analyses of the effect of rs1045411 on the outcome of GC patients. Stratified analyses of the associations between genotypes of rs1045411 and OS of GC patients by age, Lauren classification, differentiation, TNM stage and ACT(2A),and the effect on RFS of rs1045411 AG/AA genotypes in the adverse groups (2B). Patient numbers may not add up to 100% of available subjects because of missing genotyping data.

We conducted stratified analyses to evaluate the associations between genotypes of rs1045411 and OS of GC patients by age, Lauren classification, differentiation, TNM stage and ACT. The significant protective effects conferred by rs1045411 was more prominent in adverse subgroups with a HR range from 0.39 to 0.69 (Fig 2A). For details, the significant decreased death risk associated with the variant-containing genotypes (AG/AA) of rs1045411 was observed in older patients (HR = 0.69, 95%CI: 0.48–0.99), diffuse type (HR = 0.67, 95%CI: 0.47–0.95), poor differentiation (HR = 0.66, 95%CI: 0.45–0.95), clinical stage III and IV (HR = 0.58, 95%CI: 0.37–0.93) and without ACT (HR = 0.39, 95%CI: 0.20–0.76). Furthermore, the present stratified analysis showed a similar trend for RFS with a HR range from 0.44 to 0.79 (Fig 2B). The results indicated that AG/AA genotypes of rs1045411 conferred the more favorable prognosis in the adverse groups.

### Joint effect between rs1045411 and Lauren classification, differentiation, stage or ACT on OS

Previous studies have shown that both genetic variations and clinical characteristics interact to play critical roles in GC progression [17]; and that where interactions do exist, the effects of clinical elements on tumor progression will be modified by genotypes. Therefore, a joint analysis was performed to assess the potential modulating effect of rs1045411 in these clinical characteristics (Lauren classification, differentiation, stage and ACT) representing status of tumor progression on OS of GC patients. As shown in Table 2, there was a significant interaction between the genotypes of rs1045411 and Lauren classification, differentiation, stage or ACT (all *P*_interaction_ < 0.001). Compared to individuals carrying AG/AA genotypes and intestinal type, those with GG genotype and diffuse type had a significantly increased death risk (HR = 2.09, 95%CI: 1.42–3.08, *P* < 0.001). The similar results were also found in patients carrying GG genotype with poor differentiation (HR = 2.48, 95%CI: 1.70–3.62, *P* < 0.001), in patients with GG genotype of stage III/IV (HR = 2.99, 95%CI: 2.04–4.38, *P* < 0.001), and in patients carrying GG genotype with ACT (HR = 4.49, 95%CI: 2.70–7.45, *P* < 0.001) in comparison with the corresponding reference group.

**Table 2:** Joint effect of rs1056560 genotypes and Lauren classification, differentiation, stage, chemotherapy on OS.

Variables	Death/Total^a^	HR (95%CI)^b^	*P*-value^c^
AA/AG + intestinal	57/153	Reference	
GG + intestinal	119/287	1.19 (0.80–1.79)	0.391
AA/AG + diffuse	90/198	1.40 (0.91–2.16)	0.216
GG + diffuse	201/363	2.09 (1.42–3.08)	**< 0.001**
*P*_interaction_			**< 0.001**
AA/AG + Well/Moderate differentiation	66/184	Reference	
GG + Well/Moderate differentiation	137/338	1.28 (0.88–1.86)	0.198
AA/AG + Poor differentiation	81/172	1.63 (1.06–2.50)	**0.025**
GG + Poor differentiation	180/316	2.48 (1.70–3.62)	**< 0.001**
*P*_interaction_			**< 0.001**
AA/AG + Stage I/II	96/249	Reference	
GG + Stage III/IV	189/458	1.12 (0.82–1.52)	0.482
AA/AG + Stage I/II	59/113	1.74 (1.11–2.73)	**0.015**
GG + Stage III/IV	135/207	2.99 (2.04–4.38)	**< 0.001**
*P*_interaction_			**< 0.001**
AA/AG + With chemotherapy	75/203	Reference	
GG + With chemotherapy	176/374	1.52 (1.07–2.52)	**0.020**
AA/AG + Without chemotherapy	30/55	2.05 (1.12–3.74)	**0.019**
GG + Without chemotherapy	79/101	4.49 (2.70–7.45)	**< 0.001**
*P*_interaction_			**< 0.001**

Note: HR indicates hazard ratio; CI, confidence interval.

^a^ Numbers may not add up to 100% of available subjects because of genotyping fail.

^b^ Adjusted by age, sex, Lauren classification, differentiation, TNM stage, and chemotherapy where appropriate.

^c^ The significant values were shown in boldface (*P* < 0.05).

### Functional effects of rs1045411 on gene expression

**Fig 3:**
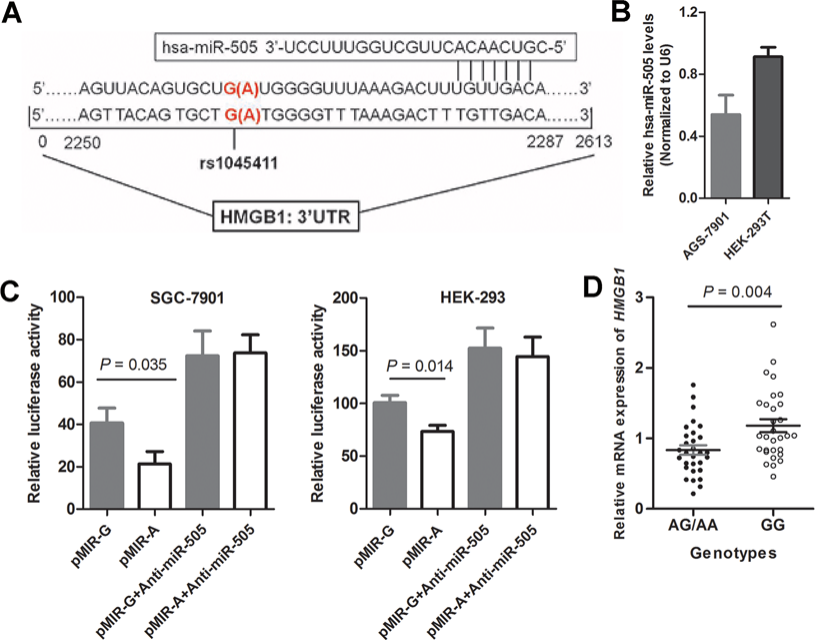
Functional effect of SNP rs1045411 genotypes on gene expression by luciferase reporter assay. (A) The sequence including SNP rs1045411 in 3’UTR of *HMGB1* gene. (B) Relative expression levels of hsa-miR-505 in SGC-7901 and HEK-293T cells. (C) The effect of SNP rs1045411 genotypes on the expression of *HMGB1* gene in SGC-7901 and HEK-293T cells. (D) Relative mRNA expression level of *HMGB1* gene in 60 GC tissues with different SNP rs1045411 genotypes. Recombinant vector (pMIR-rs1045411-G or pMIR- rs1045411-A) and pTL-TK were co-transfected into SGC-7901 and HEK-293T cells. Data are expressed as mean ± standard deviation (SD) of three independent experiments. Student’s *t* test was used to examine statistical difference.

Bioinformatics analysis (http://www.microrna.org/microrna/home.do) revealed a close proximity of tag SNP rs1045411 to the predicted microRNA binding sites (hsa-miR-505) in the 3’-untranslated region (3’UTR) of *HMGB1* gene [22] (Fig 3A). We first confirmed the expression of miR-505 in SGC-7901 and HEK-293T cell lines, and found that miR-505 had a relatively higher expression level (Fig 3B). To investigate whether the genotypes of tag SNP rs1045411 in the 3’UTR of the *HMGB1* gene could alter gene expression, two cell lines were transfected with luciferase reporter plasmids containing either the wild (GG) or variant (AA) genotype of SNP rs1045411 (Fig 3C). The results demonstrated that SNP rs1045411 significantly showed an effect on the normalized luciferase activity in both transfected cells. Compared to cells transfected with constructs carrying wild genotype (GG) of SNP rs1045411, cells transfected with constructs carrying variant genotype (AA) exhibited a significant decreased luciferase activity. The effect of anti-miR-505 on the luciferase activity of reporter plasmids was also evaluated in the study. The results showed that the luciferase activity of two UTR constructs (p-MIR-G and p-MIR-A) was significantly increased by anti-miR-505 in both cell lines with eliminated differences of luciferase activity between two reporter plasmids. Moreover, we used quantitative RT-PCR to investigate the effect of SNP rs1045411 genotypes on HMGB1 mRNA expression in 60 GC tissues (30 with GG genotype and 30 with AG/AA genotypes) from validation set. As shown in Fig 3D, we found that mRNA expression level of HMGB1 was significantly higher in the carriers with wild (GG) genotype of rs1056560 than those who carried variant (AG/AA) genotypes (1.04±0.50 *vs*. 0.79±0.37, *P* = 0.004).

## Discussion

In present study, we investigated the association of genetic polymorphisms in *HMGB1* gene with the prognosis of GC patients by two-stage analysis of training and validation sets. We demonstrated that AG/AA genotypes of tag SNP rs1045411 in *HMGB1* 3’-UTR are significantly associated with a better OS in a set of 704 GC patients when compared with GG genotypes. This significant association was confirmed in an independent validation set of 326 GC patients as well as a pooled analysis of all 1030 GC patients. Functional assay indicated that SNP rs1045411 genotypes had a significant influence on mRNA expression of *HMGB1* in GC tissues as well as two tumor cell lines. Furthermore, the favorable prognostic effect of rs1045411 was more evident in the adverse subgroups of GC patients. Additionally, the joint analysis found a significant interaction of gene-clinical elements. To the best of our knowledge, this study for the first time reported the association between *HMGB1* gene polymorphisms and GC prognosis. Once validated, *HMGB1* SNP rs1045411 may be used as a prognostic marker in combination with traditional clinical prognosis factors for the decision-making of GC individual treatment.

The increasing evidences suggest that elevated HMGB1 is associated with tumor metastasis and poor prognosis [16, 23–26], making HMGB1 an attractive tumor biomarker. It has been suggested that HMGB1 functions as a potentially oncogenic protein to promote tumor progress [15, 27, 28], and its overexpression in cancer cells affects the anti-cancer T-cell response through activation of intracellular signaling [15, 29]. Indeed, the elevated expression of HMGB1 has been detected in of patients with GC and other types of cancer [30–32], and its expression is closely associated with tumorigenesis [33], tumor invasion and metastasis [29]. Furthermore, Chung et al [34] demonstrated that serum HMGB1 levels were also significantly associated with tumor invasion, metastasis, growth, as well as poor prognosis. Collectively, these findings support an important role of HMGB1 in cancer transformation, tumor growth and invasion.

Despite the extensive investigations of HMGB1 expression in tissues and its corresponding serological activity on cancer evolution, there are a few studies focusing on the effect of the SNPs in *HMGB1* gene on cancer prognosis or treatment response so far. In a group of Chinese patients with lung cancer, two SNPs, rs141215 and rs2249825, have been associated with platinum-based chemotherapy responses [35]. Similarly, in patients with oral squamous cell carcinoma, another *HMGB1* polymorphism at the SNP rs3742305 has been associated with tumor progression and recurrence-free survival [36]. However, we excluded the SNP rs3742305 from the present study because it is in strong linkage disequilibrium with the SNP rs1045411. In present study, we found that variant-containing (AG/AA) genotypes of tag SNP rs1045411 were significantly associated with a better OS in patients with GC, supporting an important role of HMGB1 in GC evolution.

Since SNP rs1045411 is located in the 3’UTR region of *HMGB1* gene, it may influence the expression of *HMGB1* gene in GC patients. To date, however, there are no studies to evaluate the functions of the SNP rs1045411. We found that rs1045411 is in close proximity to a predicted microRNA binding site (has-miR-505) [22], suggesting that such a variation at this position may affect the stability of mRNA and binding activity to microRNA, thereby modulating gene expression [37]. Indeed, our luciferase reporter assays confirmed a significant influence of rs1045411 on the post-transcriptional regulation of *HMGB1* gene in a miR-505-dependent fashion. Moreover, by examining HMGB1 mRNA expression level in 60 GC tissue samples with genotype data of SNP rs1045411, we found that the tissues carrying variant-containing (AG/AA) genotypes had significantly decreased HMGB1 mRNA expression levels compared to those with homozygous wild (GG) genotype. Taken together, our experimental data indicated that the favorable prognostic effect conferred by variant-containing (AG/AA) genotypes of rs1045411 was closely associated with aberrant expression of *HMGB1*.

Our current study demonstrated that the significant or borderline protective effects of variant-containing (AG/AA) genotypes of SNP rs1045411 on OS and RFS of GC patients were found almost completely in the adverse (but not in favorable) strata patients. This agrees with previous studies showing that SNPs affect cancer survival more prominent in specific subgroup patients. For instance, Wang *et al*[38] have reported that *PSCA* rs2294008 is significantly associated with the survival outcomes among diffuse-type gastric cancer but not intestinal-type gastric cancer. Pu *et al*[39] have also suggested that microRNA-related genetic variants is more remarkably associated with non small cell lung cancer survival in early stage patients. We also observed a significant interaction effects between rs1045411 genotypes and clinical elements such as Lauren classification, differentiation, clinical stage, and adjuvant chemotherapy in modulating the prognosis of GC. These significant correlations suggested that SNP rs1045411 might have potential modulating roles in these clinical characteristics representing tumor progression. However, the underlying mechanisms remain to be further investigated in the future.

The present study has several distinct features. First, the enrolled patients mainly come from Shaanxi and adjacent areas, which is suitable for conducting population-based research because of the geographical stability with low mobility rate. Second, the relative large population size enrolled in present study allowed us to conduct stage-stratified analysis in training and validation sets, which limited the confounding factors of tumor and treatment heterogeneity. One major limitation of this study is that the relative small population in the validation set may result in false-negatives. Moreover, since our study was restricted to Han Chinese, we cannot rule out the generalizability issue. Future studies in larger populations and other ethnics are warranted.

Overall, as the first study observing the effect of *HMGB1* gene polymorphisms on GC prognosis in a Chinese population, our data strongly suggest that tag SNP rs1045411 in *HMGB1* gene has a significant effect on the clinical outcome of GC patients, especially for patients with advanced stage or other aggressive clinicopathological status. The present study has potential clinical significance in helping to refine therapeutic decisions in treatment of GC.

## Supporting Information

S1 Table The information and genotyping results of HMGB1 SNPs. (DOC)

S2 Table Selected demographic and clinical characteristics of gastric cancer patient population. (DOC)

S3 Table Distribution of patients' characteristics and prognosis analysis in the Training set and the Validation set. (DOC)
