# Supplementary material for: Prognostic Significance of Tag SNP rs1045411 in HMGB1 of the Aggressive Gastric Cancer in a Chinese Population
Source: PLoS One. 2016 Apr 26;11(4):e0154378. doi: 10.1371/journal.pone.0154378 (PMC4845981; doi:10.1371/journal.pone.0154378)
Supplement: S1 Table — (DOC) [file pone.0154378.s001.doc]

**S1 Table.** T**he information and genotyping results of HMGB1 SNPs.**

| NCBI SNP ID | Call rate (%) | Alleles (major/minor) | Genotyping results | | |
| --- | --- | --- | --- | --- | --- |
|  |  |  | WW | WV | VV |
| rs1045411 | 99.7 | G/A | 666 | 324 | 37 |
| rs1412125 | 99.3 | C/G | 736 | 225 | 22 |
| rs2249825 | 99.3 | T/C | 548 | 414 | 61 |
