# Supplementary material for: Prognostic Significance of Tag SNP rs1045411 in HMGB1 of the Aggressive Gastric Cancer in a Chinese Population
Source: PLoS One. 2016 Apr 26;11(4):e0154378. doi: 10.1371/journal.pone.0154378 (PMC4845981; doi:10.1371/journal.pone.0154378)
Supplement: S2 Table — (DOC) [file pone.0154378.s002.doc]

**S2 Table . Selected demographic and clinical characteristics of gastric cancer patient population.**

| Variables | Training set (n = 704) No. (%) | Validation set (n = 326) No. (%) | *P* value | Total (n = 1030)  No. (%) |
| --- | --- | --- | --- | --- |
| Sex |  |  |  |  |
| Male | 544 (77.3) | 243 (74.5) |  | 787 (76.4) |
| Female | 160 (22.7) | 83 (25.5) | 0.337^a^ | 243 (33.6) |
| Tumor site |  |  |  |  |
| Proximal | 196 (27.8) | 98 (30.1) |  | 294 (28.5) |
| Body | 257 (36.5) | 104 (31.9) |  | 361 (35.0) |
| Distal | 251 (35.7) | 124 (38.0) | 0.353^a^ | 375 (36.5) |
| Lauren classification |  |  |  |  |
| Intestinal | 293 (41.6) | 147 (45.1) |  | 440 (42.7) |
| Diffuse | 391 (55.5) | 170 (52.1) |  | 561 (54.5) |
| Unknown | 20 (2.9) | 9 (2.8) | 0.082^a^ | 29 (2.8) |
| TNM stage |  |  |  |  |
| I | 147 (20.9) | 64 (19.6) |  | 211 (20.5) |
| II | 339 (48.2) | 157 (48.2) |  | 496 (48.2) |
| III | 163 (23.2) | 74 (22.7) |  | 237 (23.0) |
| IV | 55 (7.7) | 31 (9.5) | 0.811^a^ | 86 (8.3) |
| Differentiation |  |  |  |  |
| Well | 169 (24.0) | 75 (23.0) |  | 244 (23.7) |
| Moderate | 185 (26.3) | 93 (28.5) |  | 278 (27.0) |
| Poor | 336 (47.7) | 152 (46.6) |  | 488 (47.4) |
| Unknown | 14 (2.0) | 6 (1.9) | 0.898^a^ | 20 (1.9) |
| Chemotherapy |  |  |  |  |
| No | 294 (41.8) | 159 (48.8) |  | 453 (44.0) |
| Yes | 410 (58.2) | 167 (51.2) | 0.098^a^ | 577 (56.0) |
| Relapse |  |  |  |  |
| Yes | 423 (58.4) | 218 (66.8) |  | 641 (62.2) |
| No | 301 (41.6) | 106 (33.2) | 0.006^a^ | 389 (37.8) |
| Death |  |  |  |  |
| Yes | 300 (41.4) | 182 (55.8) |  | 482 (46.8) |
| No | 424 (58.6) | 144 (44.2) | 0.001^a^ | 548 (53.2) |
| Age (years), median (range) | 57 (20 - 83) | 57 (21 - 81) | 0.793^b^ | 57 (20 - 83) |
| Follow-up time (months), median (range) | 46 (6 - 80) | 72 (6 - 89) | <0.001^b^ | 51 (6 - 89) |

Notes: TNM indicates tumor-node-metastasis; Significant P value was in bold.

^a^ The P values were calculated using a Pearson Chi-Square test.

^b^ The P values were calculated using a Mann-Whitney U test.
