# Supplementary material for: Prognostic Significance of Tag SNP rs1045411 in HMGB1 of the Aggressive Gastric Cancer in a Chinese Population
Source: PLoS One. 2016 Apr 26;11(4):e0154378. doi: 10.1371/journal.pone.0154378 (PMC4845981; doi:10.1371/journal.pone.0154378)
Supplement: S3 Table — (DOC) [file pone.0154378.s003.doc]

**S3 Table. Distribution of patients' characteristics and prognosis analysis in the Training set and the Validation set.**

| Variables | Training set (n = 704) | | | |  | Validation set (n = 326) | | | |  | Pooled analysis (n = 1030) | | | |
| --- | --- | --- | --- | --- | --- | --- | --- | --- | --- | --- | --- | --- | --- | --- |
|  | Deaths/  Total  300/704 | HR^a^ (95% CI) | Relapse/  Total  423/704 | HR^a^ (95% CI) |  | Deaths/  Total 182/326 | HR^a^ (95% CI) | Relapse/  Total 218/326 | HR^a^ (95% CI) |  | Deaths/  Total 482/1030 | HR^a^ (95% CI) | Relapse/  Total 641/1030 | HR^a^ (95% CI) |
| Age |  |  |  |  |  |  |  |  |  |  |  |  |  |  |
| ≤57 | 146/339 | Reference | 194/339 | Reference |  | 90/160 | Reference | 108/160 | Reference |  | 236/499 | Reference | 302/499 | Reference |
| >57 | 154/365 | 0.99 (0.78 – 1.26) | 229/365 | 1.14 (0.89 – 1.36) |  | 92/166 | 0.86 (0.64 – 1.31) | 110/166 | 0.95 (0.67 – 1.29) |  | 246/531 | 0.91 (0.73 – 1.28) | 339/531 | 1.08 (0.76 – 1.35) |
| Sex |  |  |  |  |  |  |  |  |  |  |  |  |  |  |
| Male | 243/544 | Reference | 336/544 | Reference |  | 136/243 | Reference | 156/243 | Reference |  | 379/787 | Reference | 492/787 | Reference |
| Female | 57/160 | 0.77 (0.57 – 1.12) | 87/160 | 0.82 (0.63 – 1.07) |  | 46/83 | 0.88 (0.62 – 1.24) | 62/83 | 0.98 (0.74 – 1.35 |  | 103/243 | 0.86 (0.60 – 1.26) | 149/243 | 0.91 (0.73 – 1.28) |
| Tumor site |  |  |  |  |  |  |  |  |  |  |  |  |  |  |
| Proximal | 91/196 | Reference | 118/196 | Reference |  | 57/98 | Reference | 67/98 | Reference |  | 148/294 | Reference | 185/294 | Reference |
| Body | 103/257 | 0.86 (0.64 – 1.17) | 149/257 | 0.93 (0.72 – 1.22) |  | 58/104 | 0.98 (0.72 – 1.26) | 68/104 | 0.99 (0.70 – 1.31) |  | 161/361 | 0.89 (0.58 – 1.32) | 217/361 | 0.96 (0.69 – 1.40) |
| Distal | 106/251 | 0.91 (0.67 – 1.22) | 156/251 | 1.09 (0.84 – 1.42) |  | 67/124 | 0.96 (0.65 – 1.38) | 83/124 | 0.97 (0.75 – 1.29) |  | 173/375 | 0.93 (0.70 – 1.27) | 239/375 | 1.01 (0.78 – 1.25) |
| Lauren classification^b^ | |  |  |  |  |  |  |  |  |  |  |  |  |  |
| Intestinal | 101/293 | Reference | 145/293 | Reference |  | 65/147 | Reference | 78/147 | Reference |  | 176/440 | Reference | 223/440 | Reference |
| Diffuse | 182/391 | **1.44 (1.02 - 1.97)** | 261/391 | **1.49 (1.05 – 2.26)** |  | 109/170 | **1.39 (1.01 - 1.96)** | 131/170 | **1.58 (1.06 – 2.36)** |  | 291/561 | **1.49 (1.08 - 2.13)** | 392/561 | **1.65 (1.09 – 2.26)** |
| Differentiation^b^ |  |  |  |  |  |  |  |  |  |  |  |  |  |  |
| Well/moderate | 122/354 | Reference | 184/354 | Reference |  | 81/168 | Reference | 97/168 | Reference |  | 203/522 | Reference | 281/522 | Reference |
| Poor | 164/336 | **1.62 (1.04 – 2.35)** | 229/336 | **1.64 (1.03 – 2.78)** |  | 97/152 | **1.55 (1.01 – 2.84)** | 116/152 | **1.83 (1.16 – 3.93)** |  | 261/488 | **1.67 (1.08 – 2.66)** | 345/488 | **1.76 (1.10 – 3.07)** |
| TNM stage |  |  |  |  |  |  |  |  |  |  |  |  |  |  |
| I | 35/147 | Reference | 59/147 | Reference |  | 26/64 | Reference | 31/64 | Reference |  | 61/211 | Reference | 90/211 | Reference |
| II | 145/339 | **1.59 (1.09 – 2.31)** | 205/339 | **1.43 (1.04 – 1.95)** |  | 79/157 | **1.45 (1.02 – 1.96)** | 89/157 | 1.41 (0.99 – 1.83) |  | 224/496 | **1.53 (1.05 – 2.23)** | 294/496 | **1.39 (1.00 – 1.89)** |
| III | 84/163 | **2.07 (1.34 – 3.20)** | 114/163 | **1.94 (1.34 – 2.79)** |  | 51/74 | **2.24 (1.26 – 3.85)** | 68/74 | **2.06 (1.29 – 4.65)** |  | 135/237 | **2.19 (1.42 – 3.38)** | 182/237 | **2.01 (1.30 – 3.15)** |
| IV | 36/55 | **2.59 (1.57 – 4.29)** | 45/55 | **2.12 (1.36 – 3.31)** |  | 26/31 | **2.91 (1.44 – 5.73)** | 30/31 | **3.29 (1.51 – 7.10)** |  | 62/86 | **2.84 (1.53 – 4.69)** | 75/86 | **2.95 (1.44 – 5.89)** |
| Chemotherapy^c^ |  |  |  |  |  |  |  |  |  |  |  |  |  |  |
| No | 58/92 | Reference | 79/92 | Reference |  | 51/64 | Reference | 58/64 | Reference |  | 109/156 | Reference | 137/156 | Reference |
| Yes | 171/410 | **0.70 (0.52 – 0.96)** | 231/410 | **0.68 (0.44 – 0.93)** |  | 80/167 | **0.77 (0.61 – 0.98)** | 96/167 | **0.73 (0.47 – 0.98)** |  | 251/577 | **0.71 (0.53 – 0.87)** | 327/577 | **0.69 (0.46 – 0.86)** |
| Note: Bold values denote P ≤ 0.05.  ^a^ Adjusted by age, sex, tumor site, tumor size, differentiation, TNM stage, and chemotherapy where appropriate.  ^b^ Other classification and unknown differentiation were censored due to the small number of subjects in this subgroup.  ^c^ Only including stage II and stage III GC patients. | | | | | | | | | | | | | | |
